# Treatment or Prophylaxis against Hepatitis B Virus Infection in Patients with Rheumatic Disease Undergoing Immunosuppressive Therapy: An Update

**DOI:** 10.3390/jcm10122564

**Published:** 2021-06-10

**Authors:** Cristina Stasi, Giacomo Tiengo, Sinan Sadalla, Anna Linda Zignego

**Affiliations:** 1 MASVE Interdepartmental Hepatology Center, Department of Experimental and Clinical Medicine, University of Florence and CRIA-MASVE Center for Research and Innovation, Careggi University Hospital, 50134 Florence, Italy; sillafelix@hotmail.it (G.T.); a.zignego@dmi.unifi.it (A.L.Z.); 2 Epidemiology Unit, Regional Health Agency of Tuscany, 50141 Florence, Italy; 3 Department of Medical and Surgical Sciences, University of Bologna, 40126 Bologna, Italy; sinan.sadalla@gmail.com

**Keywords:** hepatitis B virus infection, antiviral treatment, prophylactic treatment, rheumatic diseases, immunosuppressive therapy

## Abstract

Chronic hepatitis B virus (HBV) flares or reactivations are serious causes of morbidity or mortality in rheumatologic patients undergoing immunosuppressive therapy. The recent insights in the pathogenesis of rheumatic diseases led to the use of new immunosuppressive therapies indicated in case of failure, partial response, or intolerance of conventional synthetic disease-modifying anti-rheumatic drugs. Based on these premises, this review examines and discusses the main rheumatologic treatments that could require the initiation of prophylactic treatment or close monitoring of occult HBV infection in patients beginning antiviral therapy at the first signs of HBV reactivation, or antiviral treatment in chronic HBV-infected patients. We searched for relevant studies published in the last five years. Studies suggested that the presence of HBV infection is common in rheumatic patients and HBV reactivation during these immunosuppressant treatments is quite frequent in these kinds of patients. Therefore, before starting an immunosuppressive therapy, patients should be screened for HBsAg, anti-HBs, and anti-HBc and, on the basis of markers positivity, they should be carefully characterized for HBV infection phases. In conclusion, screening of HBV infection in patients undergoing immunosuppressive therapy with subsequent HBV monitoring, prophylaxis or treatment consistently reduces the risk of clinical consequences.

## 1. Introduction

Flares of chronic hepatitis B virus (HBV) infection or reactivation are serious causes of morbidity or mortality in rheumatologic patients who underwent immunosuppressive therapy. Currently, the chronic HBV infection has been classified by the European Association of the Liver [1] into five phases: the first phase, namely *HBeAg-positive chronic infection*, is characterized by the presence of serum HBeAg, high levels of HBV-DNA, and persistently normal ALT associated with minimal or absent liver necroinflammation or fibrosis; the second phase, namely *HBeAg-positive chronic hepatitis*, is characterized by the presence of serum HBeAg and high levels of both HBV-DNA and ALT associated with moderate or severe hepatic necroinflammation and accelerated progression of fibrosis; the third phase, namely *HBeAg-negative chronic infection*, is characterized by the presence of serum antibodies to HBeAg (anti-HBe), undetectable or <2000 IU/mL HBV-DNA levels (only few patients present high HBV-DNA levels, but usually <20,000), and normal ALT associated with minimal hepatic necroinflammation and low fibrosis; the fourth phase, namely *HBeAg-negative chronic hepatitis*, is characterized by detectable anti-HBe, persistent or fluctuating moderate to high levels of serum HBV-DNA, and persistent or fluctuating elevated ALT values associated with hepatic necroinflammation and fibrosis; the fifth phase, namely HBsAg-negative phase, is characterized by serum negative HBsAg and positive antibodies to HBcAg (anti-HBc), with or without detectable antibodies to HBsAg (anti-HBs). This phase is also known as “occult HBV infection” (OBI), characterized by undetectable HBsAg and the presence of HBV-DNA in the liver (with detectable or undetectable HBV-DNA in the serum). When detectable, the amount of HBV-DNA in the serum is usually very low (<200 IU/mL). Moreover, based on the HBV antibodies profile, OBI may be distinguished as seropositive, when anti-HBc and/or anti-HBs are positive, or, on the contrary, seronegative, when anti-HBc and anti-HBs are negative [2].

Based on World Health Organization (WHO) guidelines for HBV prevention, care, and treatment, the current potent antiviral agents are recommended for all adults over the age of 30 years with chronic HBV infection associated with persistently abnormal ALT levels and high levels of HBV replication (HBV-DNA >20,000 IU/mL), regardless of HBeAg status [3]. The treatment is also recommended in all adults, adolescents, and children with chronic HBV infection with compensated or decompensated cirrhosis regardless of ALT levels, HBeAg status, or HBV-DNA levels. These guidelines, according to a public health approach, consider the feasibility and effectiveness of new antiviral agents that present minimal risk of resistance and a very high rate of tolerability. Several studies demonstrated that long-term complete suppression of HBV replication by nucleosides/nucleotides analogues (NUC) reduces the risk of developing liver cirrhosis [4,5], hepatocellular insufficiency, and hepatocellular carcinoma [6,7], as well as its recurrence after curative treatment of HBV-related hepatocellular carcinoma [8,9,10] and induced liver fibrosis regression [11].

Several lines of evidence [12,13] showed that the screening of HBV infection in rheumatologic patients who needed immunosuppressive therapy reduces the risk of HBV clinical consequences such as reactivation in OBI patients. 

Based on these premises, this review examines and discusses the main rheumatological treatments that require the initiation of prophylactic treatment or close monitoring of OBI patients to begin antiviral therapy at the first signs of HBV reactivation, or antiviral treatment in chronic HBV-infected patients.

## 2. Clinical Epidemiology of HBV Infection and Risk of Reactivation in Patients with Rheumatic Diseases during Immunosuppressive Therapy

The HBV infection in rheumatic patients is not an uncommon event [14]. A recent study conducted on 292 patients with rheumatic diseases who did not receive vaccination against HBV showed a prevalence of HBsAg positivity of 2% and the presence of any marker of HBV infection in 24% of cases. Moreover, of the 70 patients who tested positive for any marker of infection, 30% were unaware of their condition [15].

HBV infection in patients with rheumatic diseases should always be evaluated. Before starting an immunosuppressive therapy, patients should be screened at least for HBsAg, anti-HBs, and anti-HBc to characterize the phase of infection, according to the European Association for the Study of the Liver [1], American Association for the Study of Liver Diseases [16], and Italian consensus guidelines [17]. However, routine screening in rheumatic patients still seems to be unsatisfactory. Lin et al. evaluated retrospectively patients with rheumatoid arthritis (RA) starting a first DMARD in the United States and Taiwan. The authors found that the overall testing rate for HBV infection was 20.3% in the United States and 24.5% in Taiwan [18]. A retrospective evaluation of the National Database of Japan showed that in patients with RA, HBsAg, anti-HBs, and anti-HBc laboratory tests were performed in 28.23%, 12.52%, and 14.63% of patients, respectively, during the baseline month [19]. HBV reactivation can be divided into three phases. First, an increase in serum HBV-DNA or the presence of HBeAg in previously negative patients can be observed (replication phase). The second phase corresponds to hepatocellular damage, when jaundice and a rise in serum transaminases are detected (disease activity phase). Finally, injury resolves and there is a progressive return to baseline levels of transaminases and HBV markers (recovery phase) [20].

Chen Y et al. studied 32 patients with RA and chronic HBV infection undergoing immunosuppressive therapy with glucocorticoids [21], DMARDs, and biologics. The authors showed that chronic HBV infection was significantly associated with one-year disease progression and with failure to achieve the therapeutic target at 6 months. HBV reactivation occurred in 34% of patients during the first year of follow-up. Prophylaxis with lamivudine, adefovir, or entecavir was initiated in 14 patients. In this group, 4 patients developed HBV reactivation, and 2 of them also developed hepatitis flare. Of 18 patients without antiviral prophylaxis, 7 experienced HBV reactivation and none suffered from hepatitis flare.

## 3. Occult HBV Infection in Patients Treated with Rheumatologic Drugs

In the last 20 years, new insights in the pathogenesis of rheumatic diseases led to the discovery and experimentation of various targeted molecules, indicated in case of failure, partial response or intolerance towards conventional synthetic disease-modifying anti-rheumatic drugs (csDMARDs). Currently, it is possible to distinguish between two categories of drugs. Biotechnological drugs (bDMARDs) consist of complex molecules, expressed by engineered cell lines, generally represented by whole monoclonal antibodies or their fragments joined to specific proteins. The second category consists of targeted synthetic anti-rheumatic drugs (DMARDs), which usually are small molecules with defined structures designed to interact with specific key proteins. 

Recently, Harigai et al. [22] investigated the incidence and risk factors of HBV reactivation in patients with anti-HBc antibodies treated with baricitinib for RA [21]. In this study, a total of 2890 patients received at least one dose of baricitinib in the phase 3 clinical trial. Of these 215 patients with baseline positivity for anti-HBc, 4 patients present reactivation of HBV. Based on literature data, the risk of HBV reactivation in HBsAg-negative/anti-HBc-positive patients is low [23].

Although the majority of international literature on this field including authoritative guidelines [1,16,24] states that patients with chronic infection have a higher risk of reactivation and flare than occult infection, they also highlighted that an important risk factor for HBV reactivation is represented by the type of the immunosuppressive drug used and then by the degree of immunosuppression, suggesting a need for HBV screening in patients with rheumatic diseases undergoing immunosuppressive drugs and monitoring or prophylaxis in occult infection.

Baricitinib, approved for RA treatment, is a potent inhibitor of JAK1 and JAK2 with a 100-fold affinity for JAK1 and JAK2 over JAK3; therefore, it also has some activities on IL-3 and IL-5 [25]. This class of molecules targets the signal transduction mechanism called Janus kinase-signal transducer and activator of transcription (JAK-STAT) pathway that transmits signals of many cytokines involved in the pathogenesis of numerous immune-mediated diseases.

According to Harigai et al. [22], another study evaluated the risk of HBV reactivation in RA patients with negative HBsAg and anti-HBs positivity and/or anti-HBc positivity treated with corticosteroids (≥5 mg prednisolone or its equivalent dose), DMARDs and/or bDMARDs, [12]. Considering the following scoring system—HBV reactivation risk score = 1 × (age >70 years) + 2 × (HBcAb positivity alone) + 1 × (treatment other than methotrexate monotherapy)—the authors found that patients with the highest score had an odds ratio of 13.01 for HBV reactivation, compared to those with the lowest score.

Corticosteroids suppress T-cell cytotoxic function, thus diminishing the host’s immune response [26]. Methotrexate (MTX) is a structural analogue of folate, which interferes with the synthesis of purine and pyrimidine nucleosides. However, the anti-inflammatory effects seem to be mediated via other pathways, mainly the activation of aminoimidazole carboxamide nucleotide transformylase, leading to increased levels of adenosine. [27]. The risk of HBV reactivation in HBsAg-negative/anti-HBc-positive patients treated with corticosteroids or methotrexate is low [23].

Schwaneck et al. evaluated the HBV reactivation in anti-HBc-positive patients treated with immunosuppressants afferent to a German tertiary rheumatology center [28]. Of 1317 patients treated between 2008 and 2017 with bDMARDs and 737 with csDMARDs, 86 had a history of HBV infection (anti-HBc positive), of whom 2 were HBsAg-positive patients. The authors compared the cohort of anti-HBc-positive patients without reactivation with those with reactivation that included more patients treated, showing that the median of anti-HBs titer and a history of three or more classes of immunosuppressants increase the risk of HBV reactivation. In this study, patients with low anti-HBs titers experienced more frequent HBV reactivation than those with high anti-HBs titers (>28 mIU/mL), suggesting prophylactic antiviral therapy in these anti-HBc-positive patients with low anti-HBs titers under intensive immunosuppressive treatments.

Kuo et al. retrospectively examined 134 different HBV serostatuses patients who received Rituximab (RTX) therapy, administered for a mean of 5.7 cycles in combination with methotrexate or glucocorticoids [29]. Rituximab (RTX) is an anti-CD20 chimeric antibody with human IgG1 immunoglobulin constant regions and variable regions from anti-CD20 murine antibody-binding CD20+ cells, inducing cellular death by antibody-dependent cell-mediated cytotoxicity, complement-mediated cytotoxicity antibody-dependent phagocytosis, and direct effects of RTX-CD20 interaction, thus leading to B-cell depletion [30,31]. This confers a moderate risk of reactivation in HBsAg-negative/anti-HBc-positive patients [23]. Of the 134 patients afferent to Dalin Tzu Chi Hospital, who were retrospectively evaluated by Kuo et al. [29] from January 2000 through December 2017, 50 patients were enrolled. This study demonstrated HBV reactivation in four patients (8%) during the 1–4 years after they received the first dose of RTX. Hepatitis flare-up occurred in two out of four patients, and one of them died. The authors compared the reactivation that occurred in the cohort of HBsAg-negative patients and HBsAg-positive patients, demonstrating a moderate risk of reactivation, significantly higher in the first cohort compared to the second one (30% vs. 4%). Moreover, all of the three patients who experienced reactivation were previously treated with adalimumab, a recombinant human tumor necrosis factor-α (TNFα)-specific monoclonal IgG1 antibody [32]. HBV reactivation was found in 100% of HBsAg-negative patients compared with 39% of HBsAg-positive patients. The authors concluded that the close follow-up of these patients, based on viral load and HBsAg or prophylaxis with an antiviral therapy, could be considered [29].

According to Kuo et al. [29], Chen et al. longitudinally evaluated 157 RA patients undergoing RTX therapy, of whom 103 (65.6%) were HBsAg negative and anti-HBc positive [33]. At baseline, before RTX treatment, 103 HBsAg-negative and anti-HBc-positive patients were stratified on the basis of the presence or absence of anti-HBs antibodies. Out of 103 patients, 20 (19.4%) were anti-HBs negative, while 83 (80.1%) were anti-HBs positive. Among the cohort of anti-HBs-negative patients, 5/20 (20%) developed HBV reactivation, while among the cohort of anti-HBs-positive patients, 4/83 developed HBV reactivation after RTX treatment. This study demonstrated that the baseline positivity for anti-HBs was a protective factor for HBV reactivation in HBsAg negativity and anti-HBc positivity in patients treated with RTX.

Among the recent studies, the Italian study by Varisco et al. demonstrated a low risk of reactivation in occult infection, examining 33 RA patients who were HbsAg-negative and anti-HBc-positive patients with undetectable HBV DNA who underwent a median of three cycles of RTX over 34 months in association with DMARD without prophylaxis [34]. Only one patient (3%) showed low serum HBV DNA levels (44 IU/ml) after 6 months of RTX treatment and was treated with lamivudine before the hepatitis flare. None of the patients seroreverted to HBsAg during RTX treatment, but six patients (21%) showed a decrease in protective anti-HBs levels and two of them became anti-HBs negative. In total, 14 patients were followed up for 18 months after RTX discontinuation and no patients experienced HBV reactivation.

Carlino et al. retrospectively evaluated data from 486 patients affected with RA treated with DMARDs [35]. Of these, 110 (22.6%) patients had an occult infection, while 376 (77.4%) were negative. No patients underwent prophylaxis with antiviral drugs.

Tocilizumab and abatacept were significantly more prescribed as the first bDMARD in RA patients with OBI and MTX were given significantly less. The study by Carlino et al. [35], for the first time, evaluated whether OBI might influence the effectiveness of first bDMARD therapy in RA patients, showing that OBI-positive RA patients had a significantly lower drug survival rate than OBI-negative patients. This was due to an impairment in clinical response to drug survival by effectiveness, but not to an increase in adverse events.

Tocilizumab is a humanized monoclonal antibody (Mab) that binds to both soluble and membrane receptors of Interleukine-6, inhibiting the proinflammatory effects of this cytokine [36,37], whilst abatacept is a fusion protein constituted by the extracellular domain of human cytotoxic T-lymphocyte-associated antigen 4 (CTLA-4) and the Fc portion of human IgG1. It acts as an inhibitor of antigen-presenting cells’ (APC) T-cell activation, through the blockade of the co-stimulatory second signal elicited by the interaction of CD80/CD86 and CD28. Moreover, abatacept alters the B-cell selection, depleting self-antigen-specific memory B cells [38,39]. These immunosuppressive drugs are generally considered to be at low risk of reactivation in HBsAg-negative/anti-HBc-positive patients [23].

Regarding tocilizumab treatment, Ahn et al. investigated the risk of reactivation in RA patients, particularly in those who were HBsAg negative and anti-HBc positive [40]. Out of 39 patients enrolled to undergo tocilizumab treatment, 15 were anti-HBc positive. In this study, none of the patients experienced reactivation of HBV with the use of tocilizumab. 

Matsuzaki et al. retrospectively evaluated the frequency of HBV reactivation in 1351 patients of whom 50 were HBV carriers (positive for the HBsAg), and 360 had resolved infections (positive for anti-HBc or anti-HBs) with RA in Japan [41]. HBV reactivation occurred in six cases with resolved HBV infections and four HBV carriers. 

Another Japanese multicenter, observational, prospective study investigated the incidence and risk factors for HBV reactivation over 2 years in patients with rheumatic diseases and resolved HBV infection (HBsAg negativity and anti-HBs positivity and/or anti-HBc positivity) treated with a dose of ≥5 mg/day prednisolone and/or synthetic or biological immunosuppressive drugs [42]. Among 1042 patients, including 959 with RA, HBV-DNA was detected in 35 (1.93/100 person-years) with >2.1 log copies/mL observed in 10 patients (0.55/100 person-years). The incidence of HBV reactivation with immunosuppressive therapy was 1.93/100 person-years in patients with rheumatic disease and OBI. No overt hepatitis was observed in patients showing reactivation of HBV infection.

A recent review and meta-analysis by Su et al. evaluated the effectiveness of antiviral prophylaxis for preventing HBV reactivation in 2162 patients undergoing antirheumatic therapy [43]. HBV reactivation rate varied from 55% to 5% by HBV status and treatment. The effectiveness of prophylaxis varies by HBV status and antiviral regimen used, but the meta-analysis demonstrated that prophylaxis was effective, especially in HBsAg- and/or HBV-DNA-positive patients. 

Moghoofei et al. performed a systematic review and meta-analysis to determine the prevalence rate of HBV reactivation in rheumatic patients [44]. In 30 studies, the overall estimation of the prevalence of HBV reactivation was 1.4; therefore, the authors concluded that rheumatic patients with OBI should be tightly monitored for possible reactivation.

The main recent studies on HBV reactivation in rheumatic patients with occult HBV infection during immunosuppressive therapies are summarized in Table 1.

**Figure 1:**
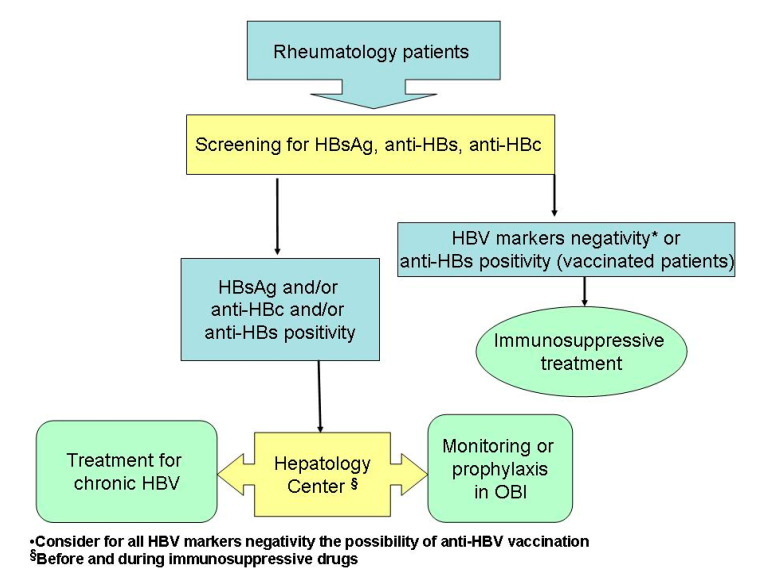
Proposed algorithm for rheumatic patients with OBI and HBV infection.

Several guidelines suggest screening for HBV (at least HBsAg, anti-HBc and anti-HBs) in all rheumatic patients before starting an immunosuppressive or hepatotoxic treatment [1,17,45]. Rheumatic patients with OBI should be referred to the hepatologist for a correct assessment of the OBI status considering both viral (including the pattern of anti-HBV antibodies) and host factors (including the anti-rheumatic therapy), with the evaluation of the opportunity that the patients should be monitored or received prophylaxis based on the risk of reactivation (Figure 1).

## 4. Chronic HBV Infection in Patients Treated with Rheumatologic Drugs

Chen et al. retrospectively evaluated the use of bDMARDs in 36 HBsAg-positive patients affected by RA. In total, 22 patients were treated with DMARDs and biotechnological agents, while 14 had a simultaneous treatment with glucocorticoids (GC), synthetics drugs, and biotechnological agents. The use of bDMARDs, alone or added to DMARDs, did not increase the risk of HBV reactivation, while the use of combined GC, bDMARDs, and DMARDs conferred the higher risk of HBV reactivation (OR of 4.83) [46]. In a retrospective, hospital-based study, conducted in patients receiving anti-TNF therapy for immune diseases without concomitant prophylaxis, HbsAg positivity was associated with 8 times increased risk to develop liver enzyme elevation, while no such association was observed among HBsAg-negative/anti-HBc-positive patients [47]. Tumor necrosis factor-α (TNFα) is a potent inducer of the inflammatory response, a key regulator of innate immunity, and plays an important role in the regulation of Th1 immune responses [48]. TNFα-inhibitors (anti-TNFα) are a class of biologic DMARDs that act as a competitive antagonist to block soluble and membrane TNF from binding their receptors [49]. A prospective observational study in patients with RA receiving tocilizumab suggested a low risk of hepatitis B reactivation [50]. All patients were treated with three consecutive intravenous doses of tocilizumab. Seven individuals were carriers of HBsAg. Among this group, five patients had chronic HBV infection and were treated without prophylaxis, while two were treated with antivirals. Three patients without antiviral prophylaxis developed HBV reactivation with normal aminotransferases. None of these patients was taking corticosteroids, and there was no difference in methotrexate dosage between these patients and the ones with chronic HBV infection who did not manifest HBV reactivation. To our knowledge, this is the only study about patients with chronic HBV receiving tocilizumab without prophylaxis.

In a multicenter retrospective study, patients with RA and chronic HBV infection were treated with abatacept. Among these patients, 38 inactive carriers received abatacept without antiviral prophylaxis, while 9 patients received lamivudine prophylaxis. In a 24-month follow-up period, there were no episodes of HBV reactivation, and no significant differences were observed in liver function tests between inactive carriers with and without antiviral prophylaxis [51]. In a prospective pharmacovigilance study on rituximab in patients with RA, two cases of HBV reactivation were observed, one of which occurred in a patient who was already receiving antiviral prophylaxis with lamivudine and consequently shifted to tenofovir [52].

The main recent studies on HBV reactivation in rheumatic patients with chronic HBV infection during immunosuppressive therapies are summarized in Table 2.

Currently, the European Society for the Study of Liver Diseases recommends that all patients positive for HBsAg should be referred to a specialist before the start of immunosuppressive therapy [1]. However, the optimal management of chronic HBV infection without hepatitis is still under debate.

The American Gastroenterology Association 2015 guidelines propose antiviral prophylaxis in both high-risk (defined by anticipated incidence of HBV reactivation in >10% of cases) and moderate-risk patients (defined by anticipated incidence of HBV reactivation of 1% to 10% of cases) undergoing immunosuppressive therapies, while suggesting the use of routinely antiviral prophylaxis only in patients who are at low risk for HBV reactivation [24]. According to American Association for the Study of Liver Diseases (AASLD) 2018 update, HBsAg-positive patients are at high risk of reactivation [16]. Patients should receive prophylaxis before the initiation of the immunosuppressive or cytotoxic therapy with high-resistance barrier drugs (entecavir, tenofovir, or tenofovir alafenamide should be preferred). A recent meta-analysis estimated the effectiveness of antiviral prophylaxis compared to no treatment. The results showed that antiviral prophylaxis, except lamivudine, were effective in preventing reactivation, and that HBsAg-positive patients had the higher benefit [43].

In conclusion, the available data strongly suggest that all HBsAg-positive patients need to be evaluated for antiviral treatment before immunosuppressive therapy.

## Figures and Tables

**Table 1:** Main studies on reactivation in occult HBV-infected patients treated with rheumatologic drugs.

Studies, Year	Study Design	Main Inclusion/Exclusion Criteria	No of Patients/Enrollment	Interventions/Treatments	Reactivation of Hepatitis B Virus
Harigai et al., 2020 [22]	Clinical trials	Patients were excluded if they had: 1) HBsAg+, 2)anti-HBs−/anti-HBc+ (in Japan, patients could enrol if HBV DNA−) or 3) anti-HBs+ and HBV DNA+.	2890 patients Of whom 215 were HBcAb positive	Baricitinib +/− csDMARDs including methotrexate (MTX), or previous treatment with active comparators including MTX or adalimumab + MTX	4/215 patients
Fukuda et al., 2019 [12]	Multicenter prospective observational study	Patients with negative HBsAg and positive anti-HBs and/or anti-HBc were enrolled, all patients were HBV-DNA negative at entry	1127 patients	Corticosteroids, bDMARDs, and/or csDMARDs	57/1127 patients
Schwaneck et al., 2018 [28]	Retrospective study	Patients on immunosuppressive therapy were evaluated for HBV screening results at any time during their treatment course	84 patients were anti-HBc positive and HbsAg negative	Corticosteroids, bDMARDs, and/or csDMARDs	8/84 patients
Kuo et al., 2020 [29]	Retrospective study	Patients who underwent rituximab (RTX) therapy for rheumatoid arthritis (RA) who tested HBsAg negative/anti-HBc positive	50 patients were anti-HBc positive and HbsAg negative	Rituximab	4/50 patients
Chen et al., 2019 [33]	Retrospective study	Patients who underwent rituximab (RTX) therapy for rheumatoid arthritis (RA) who tested HBsAg negative/anti-HBc positive	103 patients were HBsAg negative and anti-HBc positive	Rituximab	5/103 patients
Varisco et al., 2016 [34]	Retrospective multicenter study	HBsAg-negative/anti-HBc-positive patients with rheumatoid arthritis (RA) undergoing RTX	33 patients	Rituximab combined with disease-modifying antirheumatic drugs (DMARD)	0/33 patients
Ahn et al., 2018 [40]	Retrospective study	HBsAg negative and antibody anti-HBc positive	15 patients	Tocilizumab	None of the patients experienced reactivation of HBV
Matsuzaki et al., 2018 [41]	Retrospective study	1351 patients with RA were screened for HBV	50/1351 were determined to be HBV carriers and 360 patients had resolved infections	conventional synthetic DMARDs (csDMARDs: bucillamine, cyclosporin, iguratimod, leflunomide, methotrexate, mizoribine, salazosulfapyridine, tacrolimus), bDMARDs (abatacept, adalimumab, certolizumab pegol, etanercept, golimumab, infliximab, rituximab, tocilizumab), and glucocorticoids	6/360 with resolved infections
Fukuda et al., 2017 [42]	Multicenter, observational, prospective study over 2 years	1330 patients being treatment tested for HBsAg, HBsAb, and HBcAb	1193 rheumatoid arthritis patients, of whom 1123 with resolved HBV infection; 137 rheumatic disease of whom 132 with resolved HBV infection	corticosteroids (≥5 mg of prednisolone or its equivalent dose); immunosuppressive synthetic DMARDs, namely methotrexate, leflunomide, tacrolimus, mizoribine or its equivalent and/or biological DMARDs, namely infliximab, etanercept, adalimumab, tocilizumab, abatacept, golimumab, and certolizumab pegol	Frequency of HBV reactivation was calculated to be 1.93/100 person-years

**Table 2:** Main studies on reactivation in chronic HBV-infected patients treated with rheumatologic drugs.

Studies, Year	Study Design	Main Inclusion/Exclusion Criteria	No of Patients/Enrollment	Interventions/Treatments	Reactivation of Hepatitis B Virus
Chen et al., 2016 [46]	Retrospective study	Patients with rheumatoid arthritis who tested positive for HBsAg and who were not receiving anti-HBV prophylaxis were enrolled.	36 patients who tested positive for HBsAg received biotechnological treatments without prophylaxis	glucocorticoids, synthetics drugs, and bDMARDS	30/123 patients
Chen et al., 2017 [50]	Prospective observational study	Patients with rheumatoid arthritis with inadequate response to csDMARDs	5 patients with HBsAg who did not receive prophylaxis	Tocilizumab	3/5 patients
Padovan et al., 2016 [51]	Multicenter retrospective study	Patients with rheumatoid arthritis and chronic HBV infection who were treated with abatacept	38 inactive carriers received abataceptd without prophylaxis	Abatacept	0/38 patients
Vassilopoulos et al., 2016 [52]	Prospective	Patients with moderate to severe rheumatoid arthritis with inadequate response or intolerance to at least one anti-TNF	234 patients treated with rituximab	Rituximab	2 patients developed HBV reactivation
